# Understandings of genomic research in developing countries: a qualitative study of the views of MalariaGEN participants in Mali

**DOI:** 10.1186/s12910-015-0035-7

**Published:** 2015-06-16

**Authors:** Karim Traore, Susan Bull, Alassane Niare, Salimata Konate, Mahamadou A. Thera, Dominic Kwiatkowski, Michael Parker, Ogobara K. Doumbo

**Affiliations:** Malaria Research and Training Center, DEAP/FMPOS, UMI3189, Université des Sciences, des Techniques et des Technologies de Bamako, BP 1805 Bamako, Mali; The Ethox Centre, Nuffield Department of Population Health, University of Oxford, Oxford, OX3 7LF UK; Wellcome Trust Centre for Human Genetics, Oxford, OX3 7BN UK

**Keywords:** Consent, Research ethics, Bioethics, Children, Genomic research, Mali, Malaria, Qualitative research

## Abstract

**Background:**

Obtaining informed consent for participation in genomic research in low-income settings presents specific ethical issues requiring attention. These include the challenges that arise when providing information about unfamiliar and technical research methods, the implications of complicated infrastructure and data sharing requirements, and the potential consequences of future research with samples and data. This study investigated researchers’ and participants’ parents’ experiences of a consent process and understandings of a genome-wide association study of malaria involving children aged five and under in Mali. It aimed to inform best practices in recruiting participants into genomic research.

**Methods:**

A qualitative rapid ethical assessment was undertaken. Fifty-five semi-structured interviews were conducted with the parents of research participants. An additional nine semi-structured interviews were conducted with senior research scientists, research assistants and with a member of an ethics committee. A focus group with five parents of research participants and direct observations of four consent processes were also conducted. French and translated English transcripts were descriptively and thematically coded using OpenCode software.

**Results:**

Participants’ parents in the MalariaGEN study had differing understandings of the causes of malaria, the rationale for collecting blood samples, the purposes of the study and the kinds of information the study would generate. Genomic aspects of the research, including the gene/environment interaction underlying susceptibility or resistance to severe malaria, proved particularly challenging to explain and understand.

**Conclusions:**

This study identifies a number of areas to be addressed in the design of consent processes for genomic research, some of which require careful ethical analysis. These include determining how much information should be provided about differing aspects of the research and how best to promote understandings of genomic research. We conclude that it is important to build capacity in the design and conduct of effective and appropriate consent processes for genomic research in low and middle-income settings. Additionally, consideration should be given to the role of review committees and community consultation activities in protecting the interests of participants in genomic research.

**Electronic supplementary material:**

The online version of this article (doi:10.1186/s12910-015-0035-7) contains supplementary material, which is available to authorized users.

## Background

International guidance and regulation requires that participants give informed consent to research [1, 2]. Empirical research demonstrates, however, that a number of issues and difficulties can arise when seeking to ensure participants are appropriately informed about research [3-5]. In some cases, features of the context in which research is conducted may make consent processes particularly challenging, such as when research is conducted in emergency situations [6] or in populations with low levels of literacy and limited familiarity with relevant concepts [7-9]. Aspects of specific research protocols may also prove difficult to explain: in genomic research such issues include the unfamiliar and technical research methods, the implications for participants of the complicated infrastructure and data sharing requirements, and potential consequences of future research with samples and data (the nature of which may be unknown at the time consent is sought) [10-13]. All of these factors were relevant in a MalariaGEN study which enrolled young children with parental consent into a study to identify genetic factors affecting immune responses to malaria in 11 African and two Asian countries (http://www.malariagen.net/projects/cp1).

Genomic research is a novel field and unfamiliar to many people, particularly in low and middle income settings. Despite increasing amounts of empirical research being conducted on seeking consent to research, to date there is still relatively little literature available on seeking consent in low and middle income countries, specifically in relation to genetic and genomic research [10, 12-18]. Issues about how much information about genomic studies should be provided, and how best to do so, are under-researched [19]. This is an important gap in the literature given the increasing interest in genomic research in low-income settings and in particular in Africa, with initiatives such as H3Africa (www.H3Africa.org).

Against this background, this paper reports on understandings of a MalariaGEN study conducted in Mali. Rather than seeking to assess parents’ rote recall of information provided during consent processes, this study sought to determine what understandings parents reached about the study and factors affecting these [20]. Parents’ understandings were combined with data from research staff and an ethics committee about issues arising when seeking consent to genomic research. Data were analysed with the aim of identifying specific challenges arising when providing information about genomic research to participants and potential best practices in addressing these.

### The MalariaGEN study

The MalariaGEN network uses genomic research including large-scale genome-wide association (GWA) studies to identify genetic variants that are associated with resistance or susceptibility to severe malaria [21]. The genomic data are compared between multiple malaria-endemic sites to explore changes in protective immune responses depending on the conditions of malaria endemicity. The network has study sites in 11 African malaria-endemic countries, including Mali (http://www.malariagen.net/community/locations?destination=node%2F19).

The appropriate design and conduct of consent processes for MalariaGEN research was carefully considered before recruitment began [22, 23]. MalariaGEN developed a template and guidelines for obtaining consent in consultation with researchers and ethics review committees (http://www.malariagen.net/community/ethics-governance/informed-consent). Each study site took the template and adapted it to the local context before seeking ethical approval for the study. The aim of this two-stage process was to ensure that fundamental human subject protections were preserved across all the MalariaGEN sites recruiting participants, while ensuring that the protocols were also tailored to optimize the consent process at each site.

## Methods

### Study site

This empirical study was conducted simultaneously at three sites in Mali where genomic research was being conducted: Bamako, Bandiagara and Mantéourou. Bamako is the capital of Mali with a heterogeneous population, of varying cultural backgrounds and levels of education. The paediatric cases of severe malaria recruited into the MalariaGEN study in Bamako came from across the country as it is the site of Mali’s only paediatric hospital. Controls for the MalariaGEN study were recruited from the same locations as the cases, including villages up to 150 km from Bamako. Bandiagara is a multiethnic city located 700 km from Bamako in the north-east of Mali and has been a research site of the Malaria Research and Training Center (MRTC) since 1997. The Dogon and the Fulani are the two main ethnic groups in Bandiagara. The population has varying levels of education. Mantéourou is a rural village located 800 km north-east of Bamako with a primarily non-literate Dogon and Fulani population. This village has a research site used for MalariaGEN recruitment.

### Sampling

As this research aimed to examine the range and diversity of experiences and understandings of recruitment processes for a MalariaGEN study, purposive sampling of five categories of relevant stakeholders was employed (see Table 1). Categories of stakeholders, and their role in the MalariaGEN consent process, are described below.

**Table 1:** Research participants and methods of data collection

Stakeholder	Number and gender	Method of data collection
Parents of cases	26 (male)	Interview
	21 (female)	
	5 (male)	Focus group
Parents of controls	2 (male)	Interview
	6 (female)	
Senior research scientists	2 (male)	Interview
Research assistants	- Recruitment of cases and controls, seeking consent: 2 (male) and 2 (female);	Interview
	- Processing samples and managing cases: 2 (female)	
Ethics committee member	1 (male)	Interview

Forty seven parents of cases were interviewed and one focus group was held with five fathers of cases. Cases were children aged 0–5 years with severe malaria enrolled in the MalariaGEN study. The children eligible for the MalariaGEN study presented at the Gabriel Touré hospital in Bamako with a life-threatening illness. They had frequently been given traditional medicines and/or referred from one or more health centres prior to arrival at the hospital. Diagnosing the nature of the illness (severe malaria) and identifying immediate priorities for treatment were the primary focus of the clinical team and the child’s family during admission. After a brief explanation of the study procedure, researchers collected blood samples by fingerpick for the diagnosis of malaria and complications such as anemia. After diagnosis took place and eligibility to participate in the MalariaGEN study was confirmed and consent from parents was received, cases were enrolled in the research and venous sampling for the MalariaGEN study and any further diagnostic tests required took place.

Eight parents of controls were interviewed. Controls were children who were matched in terms of age and residential location to the enrolled cases. Potential controls were identified after the matched case had been enrolled in the MalariaGEN study. The MalariaGEN study team asked parents of cases to identify possible controls from their family or neighbourhood. Controls were not ill with malaria or other excluded conditions at the point of recruitment into the MalariaGEN study. The MalariaGEN recruitment process took place at controls’ homes, following discussion with the family. The majority of controls were informed about the study by families of cases before being visited by the MalariaGEN study staff. At this time that this study was conducted, many of the cases enrolled in the study had not yet been matched with controls, and so fewer parents of controls had been enrolled in the MalariaGEN study and were eligible to take part in this research.

Two senior research scientists were interviewed. Senior research scientists invited to participate in this study included principal investigators and heads of research units with extensive experience in genomic research and seeking consent. Six research assistants were interviewed. Research assistants included MalariaGEN staff responsible for the consent processes for recruiting cases or controls, or for processing research samples or providing care to malaria cases. One ethics committee member from the local committee responsible for reviewing genomic research studies was interviewed for this study.

### Data collection

The data collection methods for this qualitative study incorporated individual semi-structured interviews, a focus group and direct observations of consent processes, as well as collection of unpublished data including consent forms and information sheets (see Table 1). Parents of cases and controls were approached to take part in this study one to two days after their children were enrolled into the MalariaGEN study. Interviewers explained the study to parents, discussed any questions they had and recieved written consent from those who wished to take part. Participants were interviewed in a confidential setting by staff trained in qualitative research data collection, using the specific interview guide for each group. While most parents were interviewed within two days of recruitment into the MalariaGEN study, some parents of cases were interviewed within 30 days following their return home from the hospital, during the recruitment of matched controls. The focus group with fathers of cases was held in Sanakoroba, 30 km from Bamako. Semi-structured interviews with senior researchers, research assistants, and a member of the ethics committee were held in locations of the interviewee’s choice. There were also four direct observations of consent processes (two at Gabriel Touré hospital for cases and two in rural areas for controls). For parents of cases, data collection ceased when saturation was reached on the core topics of relevance to the study. For the other stakeholder categories, data collection ceased when all the members of the group who had consented to take part in the study had been interviewed.

Five interview guides were developed (separate guides were used for parents of cases of malaria, parents of controls, researchers, field staff, and an ethics committee member) [see Additional file 1]. The interview guides were written in French. Guides used with parents of cases and controls, and with field staff were translated into a local language (Bambara) by an external expert translator. Technical terms such as gene, heredity and ethnicity were translated using a lexicon from the National Institute of Local Languages. Interview guides were amended during the course of the research to address novel issues identified during iterative analysis of the collected data. The three interviewers and focus group facilitators had backgrounds in sociology, pharmacy and medicine respectively, and were trained in social science interview methods before beginning data collection. Interviewers and the focus group facilitators sought to minimize their influence on the views expressed by parents of cases and controls [24].

The interviews with parents of cases and controls, and the focus group were conducted in Bambara. Audio recordings of the interviews and focus group were translated and transcribed in French and English. A reverse translation from English into Bambara was made verbally to ensure consistency of translation. Interviews with researchers, field staff and a member of the ethics committee were conducted in French, and transcribed in French and English. The French and English transcripts were analysed by members of the research team fluent in Bambara, French and English and areas where the translations were inconsistent or ambiguous were carefully reviewed, and informed subsequent development of data collection materials and data analysis.

### Data analysis

Data collected in this study were analysed iteratively during data collection and novel relevant issues were incorporated into data collection materials as appropriate [25]. In this exploratory study, a thematic approach to data analysis was used to describe understandings of participation in the MalariaGEN study [26]. Analysis was undertaken using *OpenCode* software. KT and SB closely read the French and English transcripts respectively, and developed initial descriptive coding frameworks independently [25]. The two descriptive coding frameworks were compared and a common descriptive coding framework was developed and applied. Following the second round of deductive coding, inductive thematic codes were collaboratively developed by KT and SB, and refined in discussion with the other members of the research team [27, 28].

### Ethical considerations

The protocol was approved by the Ethics Committee of the University of Bamako Faculty of Medicine, Pharmacy and Dentistry of Mali, and the Oxford Tropical Research Ethics Committee (OxTREC Reference 15 08) before the study commenced. All participants gave their consent before being included in the study.

## Results

Results of the analyses of data collected from the five stakeholder groups are reported in this section. This section begins by discussing views about the recruitment processes for cases and controls. It then outlines parents’ differing understandings of the following four key inter-related components of the research:

1. Distinguishing between research and medical care
2. Aims of the MalariaGEN study
3. Future uses of blood samples
4. Using blood samples in genomic research.

### Recruitment processes

Cases were enrolled to the MalariaGEN study shortly after arrival at the hospital, at which time their parents or guardians often had limited interest in the consent process because of concerns about the health of their child. A parent of a child with severe malaria noted:

*When they explained to me the contents of the paper [the information sheet for the MalariaGEN study], I think that we were all very anxious for the child…I was asked whether I understood what was said, and so that he would not have to begin again, I said I understood very well, so he continued, and I did not understand so well, because I saw only the health of my child in the moment. That’s why I did not want to ask too much. (Parent of severe case, male).*

While there were informal opportunities to discuss the research with the study staff while the child remained in hospital for treatment, cases’ parents suggested that a second consent process for the study would be valuable once their child’s condition had been stabilised. A parent of a child with severe malaria suggested:

*The best moment to give the explanation to people is when the patient is recovered… It seems to me that one cannot enroll the child directly without giving explanation about informed consent, which is why it is given while the child is still ill. Therefore, in my opinion, one should explain informed consent at the beginning and then, when the child starts to recover, one can go over the explanation again, so that the parents have a chance to understand it when they are not distressed about their child. This way they understand it well. (Parent of severe case, male)*

In contrast, recruitment of controls took place at their homes. More time was available for explaining and discussing the research, and there was an absence of medical conditions requiring immediate treatment. Upon arrival, the research team typically received a warm welcome and expressions of gratitude for having treated the matched case, who was a family member or neighbour of the control. Illustrating this perspective, one parent welcomed the research team saying:

*“Our parents told us all the good that you have brought to them in Bamako, we were looking forward your arrival, may God reward you” (Parent of control, observed consent process).*

After identification of potential controls, researchers provided a detailed explanation about the study, based on the information sheet and consent form, before asking if there were questions about the research. This consent procedure took place in a peaceful environment where all parties could consider the information provided and parents of controls did not have suggestions for improving the consent process.

#### Distinguishing between research and medical care

As might be expected in a study in which severely ill participants are treated for their condition at no cost, many cases’ parents understood the primary objectives of the MalariaGEN study to be therapeutic care and charitable assistance to the poor. As one parent of a child with severe malaria observed, “It is to take care of children and to protect them.” (Parent of severe case, female)

In such cases participation in the research was seen primarily as an opportunity to access quality care at little or no cost, as one father described:

*When we arrived at the Gabriel Touré we found these people, they said there is a project, if we take part, the child will be treated free of charge…It is a way to help the poor people…I really liked it; I agreed to join the project. We ask them to take even more people, it will help to fight against malaria” (Parent of severe case, focus group participant, male).*

In discussions about the purpose of the research, health was prioritized by the parents of cases. The research was often not perceived as an activity generating generalizable knowledge, but as aiming to fight malaria in a particular patient through appropriate diagnosis and treatment. Illustrating this perspective one parent discussed perceived purposes of blood-taking:

*Taking blood to know if the child doesn’t have something else, for example the child who has pneumonia, if you give him a treatment of malaria without analysis, the pneumonia continues its work. (Parent of severe case, male)*

The recruitment of controls in the community was also frequently perceived not as research but as a sign of respect or a courtesy to the control’s parents, a public health intervention, or a check of the status of the case. Parents of severe cases described the recruitment of controls in the following ways:

*They came here, they gave medicines to some children, and then they sent us rice (Parent of severe case, focus group participant, male).*

*The work went well. This is their third time to come and to see if the child [case] is still doing well” (Parent of severe case, focus group participant, male).*

### Aims of the MalariaGEN study

Where parents of cases and controls did distinguish between research and the provision of care during the MalariaGEN study, a range of different views were expressed about the aims of the study. Some parents were not clear about why the study was being conducted, or described aims in general terms, such as seeking novel treatments or vaccines for malaria. There are a wide range of local views about potential environmental and behavioral causes of malaria, and given these, other parents described the study in terms of definitively identifying causes of malaria so that it could be better prevented and treated. A few parents’ descriptions focused on how the study might discover why some children get malaria and others don’t (including, in some cases, discovering inheritable characteristics which impact on susceptibility):

*They are searching a medicine to fight malaria, so it is necessary to take child’s blood that has malaria, to find a vaccine against malaria. (Parent of severe case, female)*

*They will take the child’s blood to know why she had serious malaria, figure out if it is inherited from his parents, or if he got it through other things such as food, then they will go see other children in our neighborhood to see why some children get severe malaria, while other do not. (Parent of severe case, male)*

*There are some people who have the malaria in their ethnicity while others don’t have the malaria. They want to understand why some children develop the malaria whereas others don’t develop it. Therefore they will come with the vaccine to fight the malaria. (Parent of control, female)*

### Future uses of blood samples

In this research context, there are many rumours that blood collected during studies is sold, so recruiters made very precise comments on this topic when explaining the research to parents. Most parents of cases and controls accepted that the blood collected during the study would not be sold before receiving further explanations about the future uses of the blood samples: *“The blood is not for sale, this is for disease diagnosis only” (Parent of control, female).*

In our research, participants were prompted to discuss what uses they thought would be made of the blood samples collected for the MalariaGEN study. In line with views that children were solely receiving healthcare and not taking part in research, some parents of cases thought that blood was drawn by researchers solely for diagnosis and treatment of their child. One focus group participant commented:

*My opinion is that this blood is put in the machine to know the appropriate drug to use to treat the child’s illness (Parent of severe case, focus group participant, male).*

At the point of enrolment, the boundary between research and curative medicine was by no means clear. Before enrolling a child in the MalariaGEN study, preliminary tests were made, including a thick smear to diagnose malaria, and assessment of hemoglobin and anemia. Some cases of anemia diagnosed on arrival were transfused, followed by a rapid improvement of the condition of the children. For the parents of these children, the initial collection of blood was seen as a crucial step to determine the appropriate treatment for their children, which sometimes led to the belief that all blood testing would be for the benefit of their child. A focus group participant suggested:

*Taking your child to a hospital where they will treat him without analyzing his blood, is like throwing a stone into the sun, you’re working blindly. That’s why I straightforwardly accepted. (Parent of severe case, male)*

Other parents of cases understood that the blood would be used for research into malaria, as well as for diagnosis:

“*They said that the blood will not be sold, it is to know how people die from malaria every year ” (Parent of severe case, focus group participant, male).*

*“It is true that the benefit of treatment is great, but the blood is collected for research so that one day the disease can be eliminated from our country, this is my way of understanding.” (Parent of severe case, focus group participant, male).*

Some parents who knew that blood would be used for research purposes, did not feel the need to discuss this further with researchers, “*Well, I think the purpose is to better understand malaria, if not I do not think that they can do something else with the blood ”(Parent of severe case, male).* Others did not express views about the future research uses of the blood samples “*No, they did not tell me anything about it*” *(Parent of severe case, male).*

While some parents of cases and of controls were comfortable with the concept that the blood would be tested for multiple diseases during the research, others raised concerns, the most common of which related to HIV research. One parent of a severe case discussed this explicitly:

*I have asked the doctor whether the blood will be used for malaria research only or also for AIDS, and he told me that it is only for malaria. I therefore told him to take the amount of blood he wants” (Parent of severe case, focus group participant, male).*

### Using blood samples for genomic research

Parents of cases and controls who discussed the use of blood samples for research purposes were further prompted to discuss genomic aspects of that research. Given the complexity of the topic, views of the staff recruiting participants were also sought, to determine their understanding of genomics and issues arising with discussing genomics during recruitment. The views of both staff and parents are discussed below.

Understandings of research among research staff differed depending on the nature of their responsibilities. Ward and field staff who were not directly involved in the consent process did not have the same level of understanding as senior researchers and staff recruiting participants. One research assistant noted:

*“I was told that the blood sample will be used for genomic research, but I do not know exactly what genomics means” (Research assistant, female).*

Findings from interviews with research staff and from the direct observation of consent procedures, indicate that staff recruiting participants had a good understanding of the genomic aspects of the research as described in the information sheet for the MalariaGEN study. However, the translation of these concepts into language accessible to participants (Bambara) posed significant problems. Consequently research staff suggested that further training and capacity development to assist in such translation would be valuable.

During recruitment, explaining genomic aspects of the study in an understandable manner proved challenging. Explanations of the genomic component of the MalariaGEN study were brief or sometimes absent during the verbal consent process for enrolling cases. Additionally, some parents of cases thought such aspects of the research were too technical and difficult to understand, or of limited interest “*Well, I think this is complicated for us*” *(Parent of severe case, male).*

In contrast, other participants discussed concepts of heredity of susceptibilities to disease from parents to children and their role in research, “*the children of sick parents will be fragile while those of healthy parents will resist disease*” *(Parent of severe case, focus group participant, male).* Additionally, an interrelationship between genes and susceptibility to a particular infection was mentioned by few participants, as outlined in the following quotation:

*It’s something from your ancestors, and some diseases can be caught from genes…I think that this is all connected to genetics. Some children come from their mother’s side; others come from their father’s side. For example, this child has come to the hospital three times for severe malaria. Some of my children rarely get sick. (Parent of severe case, male)*

Parents’ understanding of relationships between genes and malaria was further complicated by the range of views of causes of malaria, including mosquitos, a variety of different foods, insufficient hygiene and other environmental factors. It was not clear to some participants how genes could interact with all these potential causes of malaria. Parents of cases and controls illustrated this point by noting:

*They said that it was mosquitoes that cause malaria; that if they bite someone with malaria and then bite someone who isn’t sick, the mosquito transmits malaria. Even when we’re talking outside at night, you can get malaria. But here in the bush, even if you say that people aren’t going to believe you… There is also cold weather and when you bathe. Washing children at night can give them malaria. (Parent of severe case, male)*

*Malaria is caused by mosquitoes, dirty water and not cleaned houses. Mosquitoes live in dirty, stagnant water. After, they bite people, that’s what gives malaria. (Parent of control, female)*

*I’m not sure if we can get malaria from food, but mother to child transmission is possible during breastfeeding (Parent of control, female).*

The role of heritable characteristics in malaria transmission was complicated by views that children typically inherited characteristics from one parent and that maternal transmission of malaria in during pregnancy and breastfeeding was possible*.* Finally, a lack of clear inheritance patterns of resistance or susceptibility to severe malaria led to doubt that genes had any role to play in the severity of malarial infections:

*This problem of gene, I am not convinced. People get illnesses by luck; it likes some more than others. Myself, I grew up among people that are my parents, but I never went to the hospital or did an injection. But … my parents fall sick. Therefore the problem of gene, me I do not believe. (Parent of severe case, focus group participant, male)*

*You can do research and see what provokes the illness, but problems of parents or genes, umm, I don’t say it is false but I don’t say that I agree with that, because the period during which the illness [malaria] arrives. For example next to my family … this family’s children fell sick when my children were healthy, but now my children are sick and the others are healthy. That is due to God, this is not a parent or a society…there are familial diseases, hereditary disease that come by the family, but this is not for malaria. (Parent of severe case, male)*

## Discussion

In Mali, as in other African countries, efforts have been made to develop protection for participants in research, namely, the training of investigators, community involvement, and the mandatory review of research protocols by an independent ethics committee [29, 30]. Guidance and regulation on research ethics requires that consent to research must be appropriately informed if it is to be valid [1, 2, 31]. While there is some consensus about core topics that should be covered in all recruitment processes, researchers are required to determine how much information about each of these core topics should be provided, and how to support understanding of the information on a study by study basis [8, 32]. The limited empirical research into developing consent processes for genomic research suggests that promoting participants’ understanding that they are enrolled in genetic or genomic research and the implications of such enrollment can present challenges in a number of African settings [10, 12-15, 17, 18].

The findings from this study confirm that determining how much information should be provided to participants and how best to support understanding of genomic research is not straightforward. Similarly to staff recruiting MalariaGEN participants in Ghana [13], many researchers in this study reported difficulties with explaining the research in local languages, due to the lack of similar concepts in such languages. While research participants in this study overwhelmingly reported satisfaction with their participation in the MalariaGEN study, a number of issues about their understandings of the study were identified. Before understanding genomic components of the research, MalariaGEN participants needed to understand that they were participating in research, that blood samples were collected for research purposes, and that the research aimed to investigate inheritable factors contributing to why some children are more susceptible to severe malaria than others. Even in cases where these factors were understood, understanding of the genomic component of the research could remain elusive. Despite receiving consistent information about the research, parents demonstrated a range of understandings of the above aspects of the study. The implications of these differences and means of supporting understanding of each of the four aspects are discussed below.

Some parents of cases and of controls understood themselves to be consenting solely to healthcare. Although this view is often termed therapeutic misconception, it could be argued that rather than a misconception, it is a logical interpretation of participation in this study [33, 34]. For cases, collection of a small additional amount of blood for research purposes may appear insignificant to parents in the context of the lifesaving healthcare provided to their child in a hospital ward. As such, the activity undertaken by researchers was characterized entirely as healthcare and its research component not singled out by parents for separate consideration. Additionally, due to concerns about the health of their child, many parents of cases had limited interest in, or peace of mind to consider, information provided about the research, and in some cases sought to curtail the consent process.

In contrast, during recruitment of controls, there was no pressing need for healthcare and more time to discuss the study with parents and family in a familiar setting. Despite the lack of healthcare provision, and a more in depth consent process, parents of controls were very aware of the healthcare received by cases and some characterized the MalariaGEN study as a public health intervention for malaria, rather than research.

Research into therapeutic misconception demonstrates that it is pervasive, arising in relation to multiple kinds of research in a range of settings [35]. Even highly educated participants may have difficulties reconciling information provided about unfamiliar research concepts with what appears to be healthcare provided by healthcare staff [36]. Participants may also prefer to view what is being provided as individualized care based on their condition, rather than a standardized protocol being assessed to provide generalizable knowledge.

In the face of these environmental cues (such as the provision of information about research in healthcare settings), and personal hopes, identifying the best means of addressing therapeutic misconception about genomic research is challenging. It is clear that simply providing more information about the research may not be sufficient to address therapeutic misconception. Measures to improve information provision and address environmental cues in the healthcare setting, such as a two stage consent process and having research staff wear plain clothes in contrast to uniformed healthcare staff, proved of value in assisting parents of cases in distinguishing between healthcare and research during MalariaGEN research in Ghana [13]. However, such measures would not have assisted the parents of controls in this case, and further research would be valuable to determine how to minimize therapeutic misconception during genomic research in low and middle income settings.

As discussed above, when parents of cases and controls distinguished between research and care, they reported a range of understandings of the aim of the MalariaGEN study. Some perspectives specifically encompassed human genomic research, and others excluded it (such as identifying environmental causes of malaria). Care is needed to determine how best to describe the aims of research in a comprehensible fashion, particularly when complex and unfamiliar research methods are involved. Some discussions of research aims may identify misconceptions about the disease being studied (such as views that malaria can be caused by oily food or transmitted by breastfeeding) which should be addressed to enable participants to limit their exposure to future malarial infections. In other cases, participants may understand the aims of the research in very general terms, such as helping to fight against malaria, or developing treatments and vaccines. In such cases questions arise about the extent of researchers’ responsibilities to ensure that participants are aware that the research will entail comparing individual’s inheritable characteristics. These issues are discussed further below.

In this and many other African contexts, taking blood samples for research is a sensitive topic [34, 37-39]. Concerns are raised about physical and spiritual effects of taking blood, particularly when children with severe malaria have anaemia, which in local languages may be literally translated as ‘not having enough blood’ [13]. Concerns also arise about the purposes for which the blood can be used, such as being pooled with other blood samples for sale (particularly in contexts such as this where blood must be purchased from the blood bank if a donor cannot be found) or used in rituals [34]. Taking of blood for diagnostic tests during healthcare in contrast, may be less controversial, as the need to identify exactly what is wrong with a sick child is widely acknowledged.

During genomic research where healthcare is also provided, care is needed to ensure that participants, or in this case participants’ parents, are aware that some of the blood will be taken for research purposes, and what tests will be run with it. Particular care will be needed where it is likely that some tests, such as HIV tests, are likely to be of concern to participants. Some research groups are seeking to build awareness of how blood is used in research amongst local communities by giving tours of research facilities and addressing misconceptions about uses made of blood during research [15, 40]. The value of these and other approaches to build awareness of what will be done with blood samples should be reviewed in settings where genomic research is conducted.

There are a number of potential limitations to this study. There were varying intervals of time between parents of cases’ and controls’ enrolment in the MalariaGEN research and their participation in interviews or the focus group for this study. In such cases reported understandings might be impacted by abilities to recall information over time, leading to participants being reported as having less understanding of the research than they did at the point of enrolment into the MalariaGEN study. Due to recruitment of matched controls having to lag behind recruitment of cases, there were fewer parents of controls eligible to participate in this qualitative study: eight consented to be interviewed and none consented to take part in a focus group. Additionally the higher female to male ratio in the parents of controls, when compared to parents of cases, may have affected our findings. While additional data from parents of controls could have been valuable in identifying novel considerations or elements of understandings, data were saturated on core topics discussed during interviews. Focus groups are a valuable data collection method when exploring understandings of a complex topic such as genomic research and provided an opportunity to determine how participants responded to varying views of what the MalariaGEN research entailed [41]. Five fathers of cases kindly consented to take part in the focus group, additional focus groups with mothers of cases and controls, and fathers of controls could have provided further valuable insights into how understandings of the MalariaGEN were constructed.

An additional potential limitation of the study results from the multiple languages used during data collection, analysis and reporting. The research team was very aware of the potential for meanings to be lost or misconstrued during translation, so care was to use expert translators, to back translate where appropriate, and to review translated transcripts in areas where meaning was not being preserved. Discussions of some of the concepts that proved more challenging to translate comprised a valuable component of the analysis.

## Conclusions

Genomic research is an important field of research to understand and develop responses to endemic diseases in the African region. The findings from this study demonstrate that promoting understanding of the genomic nature of research requires that there has been understanding of multiple aspects of the study. Even where there is an interest in learning about the role of inheritable characteristics in susceptibility or resistance to severe malaria, the best means of explaining the interaction between inheritable characteristics and causes of disease may be difficult to identify. This complexity is exacerbated by the difficulty of discussing some of these concepts in local languages and advice from experts in such languages may be of great value in facilitating understanding of technical aspects of the research. Capacity building to support understanding of genomic research amongst research staff, and to support the development of methods to support understanding of genomic research amongst participants, is a critical component of future genomic studies. Such capacity building ought also to include the development of training and educational resources relating to practical ethics.

It should be noted that not all participants will wish to expend significant time and effort to understand the genomic nature of research, particularly if they perceive the research to be of limited risk. In situations where understanding at the time of consent are likely to be imperfect, which is the case in genomic research wherever in the world it is conducted, important ethical questions arise relating to what levels of understanding and of which core features are required for consent to be considered ‘valid’. For example, the findings above demonstrate that significant amounts of background information are required to explain the relevance of genomics in transmissible diseases, where complex gene-environment interactions are being studied and patterns of inheritance are not straightforward. Significant levels of understanding of the technical nature of the research will also be required if the implications of sharing genomic data for secondary research purposes are to be evaluated by participants. Whilst the question of what comprises valid informed consent has an empirical component and further research such as that conducted for this study is essential, the identification of good ethical consent practice in genomics research also requires careful ethical analysis and judgment. This will include a consideration of what additional policies, practices and processes may be needed to protect participants where research demonstrates that core aspects of the research are often not well understood. In such circumstances, in addition to identifying best practices in information provision for research participants, further review of the role of ethics committees and community engagement mechanisms in genomic research is critical.

## Additional file

Additional file 1:
**Online materials.**
