# Supplementary material for: Understandings of genomic research in developing countries: a qualitative study of the views of MalariaGEN participants in Mali
Source: BMC Med Ethics. 2015 Jun 16;16:42. doi: 10.1186/s12910-015-0035-7 (PMC4469103; doi:10.1186/s12910-015-0035-7)
Supplement: Additional file 1: — Online materials. [file 12910_2015_35_MOESM1_ESM.doc]

**Understandings of genomic research in developing countries: a qualitative study of the views of MalariaGEN participants in Mali.**

**Supplementary materials : interview guides**

**Evaluation ethnographique rapide du processus de consentement pour la recherche MalariaGEN au Mali.**

**GUIDE D’INTERVIEW POUR LES PARTICIPANTS A L’ETUDE MALARIAGEN**

Se présenter et demander au participant de se présenter aussi

**1**. S’il vous plait parlez moi comment vous avez été impliqué dans l’étude MalariaGEN ?

Selon la réponse, posez les questions supplémentaires suivantes

Comment avez vous entendu parler de cette étude pour la première fois?

Est-ce la première pour vous d’être impliqué dans une étude de recherche?

**2.** Pouvez-vous me parlez des informations que vous avez reçues sur la recherche avant de décider de participer à l’étude?

**a**/ informations sur le paludisme : mode de transmission, vecteur, manifestations cliniques, gravité, prévention, etc.… ;

**b**/ Information sur le but et la justification de cette étude ;

**c**/ informations sur les procédures de l’étude ;

**d**/ risques et bénéfices liés à l’étude ;

**e**/ la confidentialité ;

**f**/ le volontariat (libre de participer ou non sans subir de préjudices par rapport aux soins médicaux fournis par l’équipe) ;

**g**/ la conservation des échantillons de sang pendant une longue durée avec possibilité de faire ultérieurement les tests génétiques ;

**h**/ analyses génomiques pour comprendre comment l’organisme réagit face au paludisme, les facteurs génétiques (héréditaires) de prédisposition aux formes graves de paludisme.

**3**. Est-ce que vous avez compris les explications qu’on vous a données par rapport à ces différents points sus cités ?

**4**. Y’avait il des points ou des concepts difficiles pour vous à comprendre (génomiques, hérédité, conservation des échantillons de sang, etc.…) ?

**5**. Est-ce que vous vous êtes sentis confortables de poser des questions ou de demander de reprendre quelque chose?

**6**. Selon vous, le moment était il propice pour vous donner toutes ces informations à fin d’avoir votre autorisation ou bien vous auriez préféré un autre moment (tel qu’après la prise en charge de votre enfant) ?

**7**. Pensez vous que le temps que les docteurs (chercheurs) ont pris pour vous expliquer le consentement pourrait affecter la prise en charge correcte et rapide de votre enfant ?

**8.** que pensez-vous comme la bonne façon de donner l’information contenue dans le formulaire de consentement ?

**a**/ explication verbale ?

**b**/ lecture détaillée du formulaire

**c**/ sensibilisation publique avant le début de l’étude ?

**e**/ formation des animateurs à l’explication du contenu des formulaires de consentement ?

**f/** émissions radio pour expliquer publiquement le consentement ?

**9**. **Prise de décision** :

**a/** Pouvez-vous nous dire quelque chose sur comment vous avez décidé de participer ou à cette étude?

**b/** Avez-vous discuté de la décision avec quelqu’un? Si oui qui (guide, membre de la famille, autres)?

**c/** Qui a pris la décision?

**d/** Pourquoi vous aviez décidé de participer?

**e/** Selon vous, les informations qu’on vous avait données sur la recherche étaient elles suffisantes et compréhensibles pour vous permettre de décider de votre participation ?

**10**. S’il vous plait parlez moi comment vous vous êtes sentis au retour à la maison?

**11.** Avez-vous des propositions pour permettre une bonne compréhension des formulaires de consentement et de faciliter la procédure de son d’administration ?

**12.** Y a-t-il autre chose que nous avons omise et que vous voudriez mentionner?

Je vous remercie beaucoup de votre participation.

**English translation:**

**Rapid Ethnographic Assessment of the Consent Process for MalariaGEN Studies in Mali**

**INTERVIEW GUIDE FOR PARTICIPANTS OF MalariaGEN STUDIES**

Introduce yourself and ask the participant to introduce him self

1. Please tell me how you have been involved with the MalariaGEN study?

According to this response, pose appropriate follow-up questions.

- 1. How did you first hear about this study?
  2. Is this your first time to be involved in a research study?

1. Please describe the information that you received on this particular research before deciding to participate in the study?
   1. Information regarding malaria: mode of transmission, vector, clinical manifestations, severity, prevention, etc…;
   2. Information on the goals and justification of the study:
   3. Information on the procedures of the study;
   4. Risks and benefits associated with this study;
   5. Confidentiality
   6. Volunteerism (freedom to participate or not without submitting to prejudice by relationships with the medical care furnished by the team)
   7. Long-term storage of blood samples for the possible future use of genetic tests
   8. Genomic analyses to understand how the human body reacts to malaria, the genetic (hereditary) factors that predispose individuals to the severe clinical forms of malaria
2. Did you understand the explanations provided to you with regard to the different points just mentioned above?
3. Are there any concepts or points that are difficult for you to understand (e.g., genomics, heredity, blood sample storage, etc)?
4. Did you feel comfortable asking questions or requesting clarification of certain points?
5. In your opinion, was the time provided to give you the information and request your authorization ideal? Or would you have preferred another moment in time (such as after the treatment of your child)?
6. Do you think that the time the doctors (researchers) took to explain the consent to you could have affected correct and timely treatment of your child?
7. What do you think about the best way to inform participant on the study (disclosure of information enclosed in the informed consent)?
   1. Verbal explanation?
   2. Detailed reading of informed consent?
   3. Public sensitization before the study start?
   4. Training of staff to informed consent process?
   5. Public explanation of the content of informed consent through the radio?
8. Decision-Making
   1. Can you please describe how you decided to participate in this study?
   2. Did you discuss the decision with anyone? If so, with whom (guide, family member, others)?
   3. Who made the decision?
   4. Why have you decided to participate?
   5. In your opinion, was the information provided to you concerning the research sufficient and comprehensible so as to allow you to decide to participate?
9. Please tell me how you felt after returning home.
10. Do you have suggestions to improve the participant’s comprehension of the consent form and to facilitate the procedure of its administration?
11. Is there anything else that we have not covered and that you would like to mention? Thank you very much for your participation.

**Evaluation ethnographique rapide du processus de consentement pour la recherche MalariaGEN au Mali.**

**GUIDE D’INTERVIEW POUR LES CHERCHEURS**

Introduire la recherche et demander le consentement.

**1**. Introduction:

Je vous serais reconnaissant si nous pouvons commencer à parler un peu de vous, de votre profil; de votre formation; comment êtes vous parvenu à ce travail :

**a**- depuis quand exercez vous ce travail?

**b**- Quelle formation spécifique avez-vous reçue pour cela?

**c**- Quelle formation avez-vous reçue dans l’administration du consentement (éthique de recherche)?

**2**. Consentement éclairé:

Maintenant je voudrais vous demandez plus spécifiquement au sujet de vos expériences dans les processus de consentement en recherche.

**a**- que pensez-vous du processus de consentement ?

**b**- Pourquoi nous demandons aux volontaires de donner leur consentement pour participer à la recherche?

**c**- voudriez-vous nous parler brièvement des étapes de l’inclusion des participants dans ce nouveau projet?

**3**. Le processus de consentement en pratique

Maintenant si nous pouvons voir spécifiquement ce qui se passe pendant la procédure d’inclusion des participants à la recherche.

**a**- Pouvez vous me parler des différentes étapes du consentement depuis le premier jour où la personne a entendu parler de la recherche jusqu’à son inclusion?

**4**. Information et compréhension

**a**- pouvez vous me parlez du processus de consentement lorsque vous rencontrez les participants ou leurs familles ?

**b**- comment décrivez-vous la recherche?

**c**- Quelle information sur la recherche semble la plus intéressante pour les participants?

**d**- Quelle information est la plus importante à fournir aux gens qui pourraient être inclus dans la recherche ?

**e**-Y a-t-il d’autres informations que vous pensez être bonnes à fournir?

**f**- Quelle est la meilleure manière de fournir ces informations?

**g**- Quelle utilité ont les fiches d’information et du formulaire de consentement éclairé?

**h**- En pratique, que pensez-vous du niveau de compréhension des participants sur la recherche?

**i-** selon vous, quels aspects de la recherche sont ils faciles à comprendre pour les participants?

**j**- selon vous, quels aspects de la recherche pensez-vous qui sont difficiles pour eux à comprendre?

**k**- Que faites vous pour les aider à comprendre (formation, discussion entre le personnel de soutient, pratique, explication de la recherche génomique)?

**l**- En pratique y a-t-il de variation dans le contenu de l’information fournie aux participants?

Si oui, quel est son impact?

**5**. Motivation / volontariat

Si nous pouvons examiner spécifiquement les motivations et le désir de participation à la recherche.

**a**- Selon vous, quelles sont les motivations qui amènent les volontaires à prendre part à la recherche?

**b**- Pensez vous que les volontaires comprennent de façon claire qu’ils ne sont pas obligés de participer à la recherche et qu’ils peuvent suspendre leur participation s’ils le souhaitent?

**c**- En pratique, est il facile pour un volontaire de décider de ne pas participer à une étude ou à une recherche (i- effet des bénéfices de la recherche; ii- l’effet d’implication d’autres personnes dans le processus de la prise de décision; iii - rôle des chercheurs, personnel de soutient, membres de la famille, les leaders communautaires, etc.….) ?

**6**. Questions pour conclure:

**a**- avez-vous des suggestions pour l’amélioration du processus de consentement ?

**b**- Y a-t-il quelque chose que nous n’avions pas soulignée et que vous voudriez mentionner?

Je vous remercie

**Evaluation ethnographique rapide du processus de consentement pour la recherche MalariaGEN au Mali.**

**GUIDE POTENTIEL D’INTERVIEW POUR LES MEMBRES DU COMITE D’ETHIQUE**

Introduction et remerciement pour la participation

Une explication brève de ce qui passera dans l’interview

Encourager le participant à donner des réponses détaillées

**1**. a- s’il vous plait pouvez-vous parler de votre expérience dans la revue des protocoles?

**b**- quels types de protocoles avez-vous passé en revue (essais cliniques/études génomiques, etc..)?

**c**- avez-vous une expérience avec les protocoles de recherche en génomique?

**d**- selon vous, quelle peut être la responsabilité de la communauté en recherche sur la génomique?

**2**. je voudrais vous poser des questions sur le processus de consentement.

**a**- que pensez-vous sur la facilité pour le participant à comprendre le consentement?

**b**- à votre avis, quelle est la bonne manière pour mieux expliquer le concept de la génomique aux participants?

Quelles informations aimeriez-vous inclure dans le consentement d’une’ étude génomique?

**c**- quel est le processus le plus approprié pour la compréhension des participants dans l’administration du formulaire de consentement dans les études génomiques?

**3**. Questions pour conclure

Y a t il quelque chose que nous avons omise et que vous voudriez mentionner au sujet de la recherche et le consentement?

Je vous remercie beaucoup, ça été très utile.
